# Small extracellular vesicles derived from plasma and the progression of Alzheimer's disease: insights into synaptic dysfunction and neuroinflammation

**DOI:** 10.1002/alz.088751

**Published:** 2025-01-09

**Authors:** Rishabh Singh, Sanskriti RAI, Prahalad Singh Bharti, Prasun Chatterjee, Saroj Kumar

**Affiliations:** ^1^ All India Institute of Medical Sciences, New Delhi, New Delhi India

## Abstract

**Background:**

Alzheimer's disease (AD) is a neurodegenerative disease characterized by Aβ plaques and neurofibrillary tangles, with chronic inflammation and synaptic dysfunction playing a significant contributor to disease progression and cognitive decline. Small extracellular vesicles (sEVs) are implicated in AD progression by facilitating the spread of pathological proteins and inflammatory cytokines. This study investigates the role of plasma‐derived sEVs (PsEVs) in synaptic dysfunction and neuroinflammation and their association with amyloid‐β and tau pathologies in AD progression.

**Method:**

A total of 45 [15 each in AD, mild cognitive impairment (MCI), and age‐matched healthy control (AMC)] subjects were recruited, and written informed consent was obtained (Ethics Ref No: IECPG‐670/25.08.2022). PsEVs were isolated using a chemical precipitation method, and their morphology was characterized by transmission electron microscopy. The size and concentration of PsEVs were determined using nanoparticle tracking analysis (NTA). Antibody‐based validation of PsEVs was done using CD63, CD81, TSG101, and L1CAM antibodies. Synaptic dysfunction and neuroinflammation were evaluated with Synaptophysin, Glial Fibrillary Acidic Protein (GFAP), IL‐1β, and TNF‐α antibodies. AD‐specific markers, Aβ‐42, and pTau were examined using Western blot and ELISA.

**Result:**

Our findings reveal higher concentrations of PsEVs in AD and MCI compared to AMC (p<0.0001). Aβ42 and pTau expression are significantly elevated in MCI (AUC = 0.9711, sn=100%, sp=80%, p<0.0001; AUC = 0.9689, sn=100%, sp=93.3%, p<0.0001, respectively); AD (AUC = 1, sn=100%, sp=100%, p= p<0.0001; AUC = 1, sn=100%, sp=100%, p<0.0001, respectively). Synaptophysin exhibits decreased expression from AMC to MCI to AD (p=0.047), whereas, IL‐1β, TNF‐α, and GFAP showed increased expression in MCI and AD compared to AMC (p=0.002, 0.0006, 0.0184, respectively). The increased levels of PsEVs correlate with synaptic dysfunction and neuroinflammation.

**Conclusion:**

Elevated PsEVs and upregulated Aβ42 and pTau expression show high diagnostic accuracy in AD. The decreasing synaptophysin expression and increased neuroinflammatory markers in AD and MCI patients suggest synaptic degeneration and neuroinflammation as potential indicators. These findings support the potential of sEV‐associated biomarkers for AD diagnosis and highlight synaptic dysfunction and neuroinflammation in disease progression.

